# Self-harm on a closed psychiatric ward

**DOI:** 10.1192/j.eurpsy.2022.1703

**Published:** 2022-09-01

**Authors:** N. Kool, A. Jaspers

**Affiliations:** Parnassia Groep, Centre Intensive Treatment, The Hague, Netherlands

**Keywords:** self-harm, closed ward, psychiatric hospital, nonsuicidal self-injury

## Abstract

**Introduction:**

Self-harming behavior is a frequent problem seen at patients admitted to closed wards in psychiatric hospitals. People who self-harm have al higher risk of other forms of aggressive behavior as well. Little is known about prevalence and characteristics of this behavior, the preceding triggering factors and the relation with other aggressive behaviors.

**Objectives:**

To gain insights in the self-harming behavior of patients admitted to a closed ward in a psychiatric hospital.

**Methods:**

From September 2019 till January 2021 information on self-harming incidents and aggressive behavior towards others or objects, of 27 patients admitted to the closed department of the Centre Intensive Treatment (Centrum Intensieve Behandeling), has been gathered. The Self-Harm Scale and Social Dysfunction and Agression Scale were used to gather the data.

**Results:**

Twenty of 27 patients examined (74%) showed self-harming behavior. Head banging (41,9%) and self-harming using straps/ropes (30%) occurred most. Tension/stress as triggering factor was mentioned most (19,1%), followed by reliving (13,5%) and team interaction (11,8%). Self-harming behavior occurred more in evenings then during the rest of the day. No significant difference was found in the degree of aggressive behavior towards others or objects between the group of patients harming themselves and the group that didn’t.

**Conclusions:**

This study delivers insights in self-harming behavior of patients admitted to closed psychiatric departments that can be used for prevention and treatment.

**Disclosure:**

No significant relationships.

